# Using Minimum Redundancy Maximum Relevance Algorithm to Select Minimal Sets of Heart Rate Variability Parameters for Atrial Fibrillation Detection

**DOI:** 10.3390/jcm11144004

**Published:** 2022-07-11

**Authors:** Szymon Buś, Konrad Jędrzejewski, Przemysław Guzik

**Affiliations:** 1 Institute of Electronic Systems, Faculty of Electronics and Information Technology, Warsaw University of Technology, Nowowiejska 15/19, 00-665 Warsaw, Poland; konrad.jedrzejewski@pw.edu.pl; 2 Department of Cardiology-Intensive Therapy and Internal Disease, Poznan University of Medical Sciences, 60-355 Poznan, Poland; pguzik@ptkardio.pl

**Keywords:** electrocardiogram, ECG, atrial fibrillation, machine learning, feature selection, minimum redundancy maximum relevance, MRMR

## Abstract

Heart rate is quite regular during sinus (normal) rhythm (SR) originating from the sinus node. In contrast, heart rate is usually irregular during atrial fibrillation (AF). Complete atrioventricular block with an escape rhythm, ventricular pacing, or ventricular tachycardia are the most common exceptions when heart rate may be regular in AF. Heart rate variability (HRV) is the variation in the duration of consecutive cardiac cycles (RR intervals). We investigated the utility of HRV parameters for automated detection of AF with machine learning (ML) classifiers. The minimum redundancy maximum relevance (MRMR) algorithm, one of the most effective algorithms for feature selection, helped select the HRV parameters (including five original), best suited for distinguishing AF from SR in a database of over 53,000 60 s separate electrocardiogram (ECG) segments cut from longer (up to 24 h) ECG recordings. HRV parameters entered the ML-based classifiers as features. Seven different, commonly used classifiers were trained with one to six HRV-based features with the highest scores resulting from the MRMR algorithm and tested using the 5-fold cross-validation and blindfold validation. The best ML classifier in the blindfold validation achieved an accuracy of 97.2% and diagnostic odds ratio of 1566. From all studied HRV features, the top three HRV parameters distinguishing AF from SR were: the percentage of successive RR intervals differing by at least 50 ms (pRR50), the ratio of standard deviations of points along and across the identity line of the Poincare plots, respectively (SD2/SD1), and coefficient of variation—standard deviation of RR intervals divided by their mean duration (CV). The proposed methodology and the presented results of the selection of HRV parameters have the potential to develop practical solutions and devices for automatic AF detection with minimal sets of simple HRV parameters. Using straightforward ML classifiers and the extremely small sets of simple HRV features, always with pRR50 included, the differentiation of AF from sinus rhythms in the 60 s ECGs is very effective.

## 1. Introduction

Atrial fibrillation (AF) is characterized by the less organized and nearly random electrical activity of both atria accompanied by an irregular ventricular rhythm. [1,2,3]. AF can be either asymptomatic in many patients unaware of its existence or entirely symptomatic with paroxysmal or persistent palpitations, dyspnea, angina, worsened exercise tolerance, and occasional syncope [2,3]. This arrhythmia is associated with a significantly increased risk of heart failure, cognitive decline due to vascular dementia, ischemic stroke, and premature death [2,3].

AF is the most common sustained cardiac arrhythmia, particularly in older people and those with cardiovascular risk factors such as hypertension, diabetes, smoking, coronary artery disease, or obesity [2,3,4]. It is usually a consequence of structural and/or functional changes in the left atrium or both atria [2,3,4]. However, AF is not a rare finding in structurally and functionally normal left atria, e.g., in hyperthyroid disease, after alcohol consumption, or if autonomic dysregulation is present [5,6]. The estimated lifetime risk of AF is about 30%, meaning that one in three adult individuals of European ancestry may develop it in the future [2,3,4].

Signal processing and machine learning (ML) techniques enable automatic detection of AF [2,3]. According to the most recent guidelines of the European Society of Cardiology, AF can be diagnosed using an electrocardiogram (ECG) if: (1) it is present in the entire 12-lead ECG or (2) if fewer ECG leads are available, it lasts for at least 30 s. However, in many cases of transient, paroxysmal AF, long-term ECG monitoring is required to diagnose it [2,3]. Mobile ECG monitors with built-in signal processing and ML capabilities are a promising tool for this task [2,3]. However, such devices’ computational capacity and memory are limited, and real-time performance is required. It poses additional requirements on ECG processing and classification algorithms, which have to be simultaneously time- and memory-efficient and provide sufficient performance quality.

Several approaches are used in the automated detection of AF from an ECG. Some methods analyze the electrical activity of atria because no P-wave is present on the ECG during AF. QRS cancellation methods, such as average beat subtraction [7], principal component analysis (PCA) [8], independent component analysis (ICA) [9], and singular value decomposition (SVD) [10,11], are used for atrial electrical activity extraction. The processing of atrial waveform may include time-frequency analysis to determine the presence of P-waves or f-waves (fibrillatory waves) on an ECG.

However, atrial activity-based AF detection methods are sensitive to poor signal quality. Atrial waveforms on an ECG have much lower amplitude than ventricular waveforms, are less defined at the beginning and end, and may overlap with U or T waves, particularly at higher heart rates. Technical artifacts resulting from electromagnetic noise, body movements, or poor electrical skin properties also severely impact the signal. Additionally, the methods of atrial activity extraction are computationally expensive and may not be suitable for real-time use, e.g., in mobile ECG monitors.

One commonly used approach to AF detection is the analysis of heart rate variability (HRV), defined by the variation in the duration of consecutive cardiac cycles. In contrast to AF, sinus rhythm (SR) is a normal cardiac rhythm and usually is quite regular within a specific time. Measures of beat-to-beat changes in cardiac cycle duration during SR are used for physiological and clinical purposes (mainly prediction) but also in sports and psychology studies [12,13,14,15]. In general, although some variation in the duration of cardiac cycles exists in SR, its extent is much larger in AF. HRV-based methods rely on the significant and strong irregularity of the duration of the cardiac cycles in AF. The distance between R-waves of consecutive QRS complexes corresponding to the electrical activity of the right and left ventricles of the heart can be used as the length of the cardiac cycle. R-wave detection is well established, precise, and computationally efficient. The analysis of RR intervals (differences between consecutive R-wave peaks on an ECG) enables the detection of irregularity in heart rhythm. Dozens of HRV parameters [12] are derived from RR-interval time series and can be used as input features for ML algorithms.

Using too many features in ML algorithms may bring redundant information, leading to an insignificant increase in their performance with an increasing feature set or even to deterioration of the results. The same issue applies to adding HRV features in ML algorithms for AF detection since substantial overlap exists between many HRV parameters, and some may contribute similar information. Feature selection in AF detection has been studied. In a study conducted by Michel et al. [16], several approaches, including γ-metric, mean decrease in accuracy (MDA), mean decrease in Gini (MDG), and area under the curve (AUC), were used to select the most relevant HRV features from a 60 s ECG. AUC was also employed for the feature selection in [17]. Boon et al. [18] used a genetic algorithm to optimize both the selection of the classifier metaparameters and the selection of the HRV feature set from a 15 min ECG. Mustaqeem et al. [19] selected the best features for classifying 16 different cardiac rhythms using a wrapper algorithm around a random forest. In a study conducted by de Chazal et al. [20], the best features in four groups were identified using linear discriminant analysis (LDA). In the PhysioNet/Computing in Cardiology Challenge 2017 [21], where four different rhythms (AF/SR/noisy/another rhythm) had to be identified in short ECG recordings, several participants incorporated feature selection [22,23,24]. The approaches to selecting feature sets (some of which included HRV) ranged from maximal information coefficient (MIC) and maximum redundancy maximum relevance (MRMR) [22], backward elimination [23], to the reduction of the entropy [24]. Unfortunately, the detailed results of selecting features were not included in these papers. In a study conducted by Krasteva et al. (2020), signals from the PhysioNet/CinC Challenge 2017 database were classified using features from HRV, morphology analysis, heartbeat classification, principal component analysis (PCA) of PQRST and TQ, P-wave analysis, TQ-segment analysis and noise correction [25]. The HRV parameters included the percentage of successive RR intervals differing by at least 50 ms (pRR50) and the ratio of standard deviations of points across (SD1) and along the identity line (SD2) of the Poincare plots, i.e., SD1/SD2. Relative feature importance (separately in four rhythms) was investigated based on the weights of the activated neurons in a neural network. Christov et al. [26] used forward stepwise selection with the linear discrimination analysis (LDA) classifier to select the most important features in three HR ranges (<50, 50–100, >100 beats per minute (bpm)) for differentiating four rhythms (AF/SR/noisy/other) of the PhysioNet/CinC Challenge 2017 database. The features were derived from HRV, average beat morphology, and analysis of atrial f-waves. pRR50 was ranked highest in the two upper HR ranges and SD1/SD2 was second in the 50–100 bpm range. Shao et al. [27] proposed a system for AF detection in wearable devices. Thirty-one features (including some based on RR interval series) were ranked by their importance obtained from the CatBoost model. The impact of the number of features in the ML model on the Matthews correlation coefficient (MCC) scores was presented. Parsi et al. [28] used established and new HRV parameters to predict the onset of paroxysmal AF. MRMR, infinite latent feature selection (ILFS), and least absolute shrinkage and selection operator (LASSO) were used for feature selection. The accuracy in 10-fold CV (by a patient) was 97.7%. Biton et al. [29] extracted the following features from a 7–10 s 12-lead ECG: deep neural network features, morphology, HRV, and electronic medical record system (EMR) metadata. A subset of features was selected using MRMR to predict AF occurring within 5 years (59.6% sensitivity, 96.3% specificity in the test set). Zhu et al. [30] used a combined approach with MRMR, Fisher, and correlation criteria for the selection of HRV parameters for AF detection in a database containing several types of cardiac rhythms. They also studied the impact of the number of neurons in the hidden layer of the neural network on classification performance. In a thesis by Kotynia [31], MRMR was used for ranking 24 morphology and HRV features (including SDRR, pRR50, SD1, SD2, and SD1/SD2) in 10, 15, 30, and 60 s ECGs in AF/SR and AF/non-AF classification. In 10 s and 15 s segments, pRR50 had the highest MRMR rank in AF/SR classification. However, the database was relatively small (from 324 60 s segments to 5504 10 s segments). Jiang et al. [32] studied AF detection in a 24 s ballistocardiogram using several ML classifiers. They used MRMR to select the most relevant among several novel nonlinear persistent homology features and studied the impact of the number of features on classification performance. Ballistocardiogram measures rhythmic motions of the whole body caused by heart contractions and blood propelling into the aorta. The signal quality is far from optimal, not even closely comparable to an ECG. The readings and measurements are not as reliable as an ECG. However, we found the proposed methodology useful and adapted it to conduct a similar analysis for HRV parameters from an ECG [32]. Parsi et al. [33] used HRV features from 1 min and 5 min ECG segments for the prediction of ventricular fibrillation (VF) and ventricular tachycardia (VT). First, a Student’s t-test was used to eliminate features with the lowest discriminatory properties. The remaining features were ranked using MRMR and ILFS. Three classifiers were applied to predict the VT-VF event using an optimal number of features from each method (determined in the learning phase). In 1 min and 5 min segments, the best classification results in the test set were obtained using feature sets selected by MRMR (6 features in both cases) [33].

Several ML methods (classifiers) are suitable for automatic AF detection, regardless of which feature extraction method (such as HRV analysis or time-frequency analysis) is used. A threshold for a single parameter can be used for AF/SR discrimination [34]. Support vector machine (SVM) [35,36,37,38] is widely used due to its ability to fit relatively complex datasets. Artificial neural networks (ANN) [39], including deep convolutional neural networks (CNN) [40], and recurrent neural networks (RNN) [41], are also used for the detection of AF. Training the ANN classifier can take significantly longer than SVM, depending on a neural network’s size, architecture, and different metaparameters. However, deeper neural networks can fit more complex datasets. Another classification algorithm is the decision tree [38,42]. It is easily interpretable and fast to train, but its usefulness in complex classification problems is limited. The sensitivity and specificity in AF detection reported in the literature vary depending on the dataset and the methods used. We included the results from selected studies on automated AF detection in Table 1.

We aimed to study the impact of the selection of HRV parameters (features in terms of ML) employed as inputs in ML algorithms for distinguishing AF from SR. Some HRV features have been rarely or never used for this purpose.

## 2. Materials and Methods

We used one of the most effective filter-based algorithms for feature selection, i.e., MRMR [48], which has recently been relatively widely used in ML [22,29,30,31,32,33,49]. The MRMR algorithm maximizes the relevance (ability) of the set of features for correct classification and minimizes the redundancy between the features. To determine the relevance and redundancy, the mutual information between the features and between individual features and the classification output are calculated, respectively.

We identified minimal sets of one to six of the selected HRV features, allowing us to achieve the best performance of automatic AF detection. We decided to use several different, relatively simple classifiers and compare their performance to check if the effectiveness of the MRMR-based feature selection is classifier-dependent. For feature sets containing from one to six features with the highest MRMR scores, each classifier was tuned in the 5-fold cross-validation in the training set to obtain the highest accuracy. The tuned classifiers were then trained on the entire training set and validated on the whole test set. For the small sets of HRV features, we determined statistical and diagnostic measures for the automatic AF detection algorithms. To our knowledge, such research results have never been reported.

In all models, pRR50 was the most relevant HRV feature and thus was always present. To study the effects of minimal sets composed of other HRV features, we have post-hoc defined an additional study aim to explore the diagnostic properties of ML algorithms for separating SR from AF ECGs using HRV features after exclusion of pRR50. Therefore, the entire process (feature selection, metaparameter tuning, training, and validation) was then repeated after excluding pRR50 to evaluate how much it would negatively impact the performance of classifiers.

The potential application of our findings in miniature devices determines the number of HRV features and the choice of relatively simple ML algorithms for AF detection. Devices such as bio-patches, wearables, or implantable devices are critically limited by the available computational resources and the acceptable energy consumption.

### 2.1. Data Used in the Study

Two open databases were used in the study: the MIT-BIH Atrial Fibrillation Database (AFDB) [50,51] and the Long Term AF Database (LTAFDB) [51,52]. Both databases contain ECG signals, annotations of detected QRS locations, and annotations of rhythm type. Distances between two consecutive QRS complexes, i.e., RR intervals, correspond to each cardiac cycle’s duration. Each QRS complex was annotated as one of the following beats: sinus, supraventricular or ventricular, and an artifact if the noise was present instead of a QRS complex. RR intervals were annotated with the type of beat corresponding to the QRS complex at the beginning of each cardiac cycle. The AFDB database contained 23 ECG recordings with a mean duration of 10 h. In the LTAFDB database, 84 ECG recordings lasted, on average, 24 h.

### 2.2. Software Tools

We used Python programming language (version 3.9, Python Software Foundation, Wilmington, DE, USA) for all the analyses except for the MRMR algorithm, for which we used the implementation from the Statistics and Machine Learning Toolbox in Matlab (version 2021a, Mathworks, Natick, MA, USA). For classification, we used the scikit-learn Python library (version 0.24.2).

### 2.3. Splitting Data into the Training Set and Test Set

Several different methods are used to assess classification performance [53,54]. Both k-fold cross-validation and blindfold validation were performed in this study to evaluate potential data leakage problems. Data leakage occurs when random samples for training and test datasets result in very similar data, e.g., from the same patient, present in both sets, leading to an over-optimistic estimation of classification performance [55]. In many publications, recordings from the same patients as in the training set or even different segments of the same recordings are present in the validation set [35,36,43,44], which leads to very good but unreliable results. This issue is rarely discussed in publications on ML-based arrhythmia detection. It was addressed in the context of AF detection in [46], where its impact on classification metrics was demonstrated.

In [41], data from 20 out of 23 patients in AFDB were used for the training part. The 10-fold cross-validation of the model was performed on the training set, and the data from the three remaining patients were used for the blindfold validation. The reported blindfold validation performance was even higher than in the 10-fold validation (accuracy 99.77% vs. 98.51%), which can be due to the small size of the validation set. In [47], the classifier from [41] was validated on a different database (LTAFDB) than the training set (AFDB), achieving 94% accuracy. Other results can be found in [46], where the classifier was trained on RR intervals from 5 s ECG segments from AFDB and tested on the PhysioNet Computing in Cardiology Challenge 2017, achieving 96.98% accuracy.

We argue that data leakage should not be ignored as it can lead to overfitting the dataset and decrease performance when new data is introduced. Properly dividing data into a training set and a test set is crucial for reliable estimation of classification performance. We randomly selected 2/3 AFDB patients and 2/3 LTAFDB as the training set and the remaining 1/3 of the patients from each database as the test set. This way, we aimed to achieve two things: 1. data from no patient is simultaneously present in both sets, and 2. training and test sets are both large and varied. Classification metrics from the blindfold validation on the test set were compared with the 5-fold cross-validation on the training set. The test set differed from the set used for training the ML classifiers, with no patients present in both sets in the blindfold validation. Such an approach is closer to real-life ECG monitoring when the data from the tested person was not previously used for training the AF detection algorithm.

### 2.4. Data Preprocessing

Uninterrupted, non-overlapping 60 s fragments of ECG with either AF or SR were chosen for analysis, and segments containing other rhythms were discarded. We used the QRS annotations from the databases to calculate the RR intervals. ECG fragments containing both AF and SR were also excluded to limit the scope of the study to the differentiation between pure AF and pure SR. RR intervals shorter than 240 ms or longer than 3000 ms were removed to limit the impact of potentially unnoticed technical artifacts on the analysis. ECG segments in which the removed RRs lasted at least 10% (6 s) of the total segment duration (60 s) were also discarded from the study. The number of studied AF and SR segments (before and after removing some segments with artifacts) are summarized in Table 2.

### 2.5. Feature Extraction

In the HRV analysis [12,56], many signal parameters can be computed using RR intervals time series. In AF, lengths of RR intervals usually alter more than in SR. For this study, we calculated the HRV parameters from several groups. The measures reflecting differences between consecutive RR intervals or RR intervals variance include:

- pRR50 (percentage of successive differences between RR intervals greater or equal to 50 ms)—it is an example of counting statistics in which the rate of a specific event (in this case, the difference between two consecutive RR intervals of at least 50 ms) is counted;
- SD1 (standard deviation of points in the Poincare plot across the identity line)—it reflects the short-term RR variability from the Poincare plot;
- SD2 (standard deviation of points in the Poincare plot along the identity line)—it shows the long-term RR variability from the Poincare plot;
- SDRR (standard deviation of RR intervals)—it reflects the total HRV;
- RRdif = mean(|RR_n+1_ − RR_n_|) (mean of absolute differences between successive RR) − it summarizes the averaged range of differences between two consecutive RR intervals.

The relative measures of RR-interval-derived difference or variance include:

- CV = SDRR/(mean RR) (coefficient of variance)—it reflects the dispersion of the total variance around the mean;
- SD2/SD1 [57]—describes how much the long-term variance changes with the short-term variance. Another interpretation is how much the dispersions of points along and across the identity line change when compared to another. If SD2/SD1 is over 1, then the long-term HRV is larger than the short-term HRV, and vice versa;
- relRRdif = RRdif/(mean RR) (relative RRdif)—it shows the average rate of the absolute differences between successive RR normalized to the mean of all RR intervals.

The measures of relative changes between two consecutive RR intervals:

- meanSuccRat = mean(RR_n+1_/RR_n_) (mean ratio of successive RR), the interpretation of this parameter is as follows: what is the average relative change between two consecutive RR intervals in a specific ECG segment;
- SDSuccRat = SD (RR_n+1_/RR_n_) (standard deviation of ratios of successive RR), the interpretation of this parameter is as follows: what is the variability of the relative changes between two consecutive RR intervals in a specific ECG segment.

The absolute descriptors of the RR interval distribution:

- mean RR (mean of RR intervals);
- RRrange = max(RR) − min(RR).

The relative descriptor of the RR interval distribution:

- relRRrange = RRrange/(mean RR) (relative RRrange), the interpretation of this parameter is as follows: how much the range between the shortest and the longest RR interval in a specific ECG segment is larger than the mean of all RR intervals in the same ECG segment.

Some of these parameters (mean RR, SDRR, SD1, SD2, and pRR50) are widely used in the HRV analysis of long-term ECG recordings, mainly for predictive purposes [12,58]. In [59], several of these parameters (mean RR, SDRR, SD1, SD2, pRR50, CV) were listed among typical HRV features for AF detection. The computation of these parameters is straightforward and thus potentially suitable for mobile devices. In the remaining part of the paper, we refer to analyzed ECG signal segments as samples and to the HRV parameters representing them as features. Such terminology is common in ML and might help avoid confusion between the metaparameters of classifiers and HRV parameters.

### 2.6. Feature Selection

**Figure 1:**
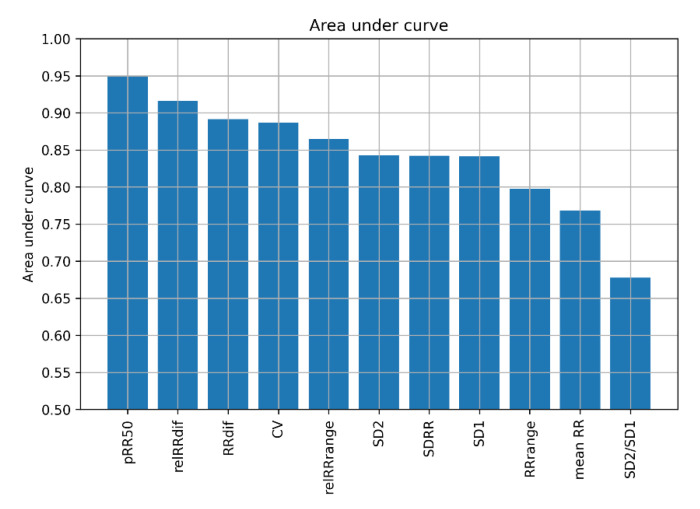
Area under receiver operating characteristic curve (AUC) of heart rate variability features. pRR50—percentage of successive differences between RR intervals greater or equal to 50 ms; SD1 and SD2—standard deviation of points in the Poincare plot across and along the identity line, respectively; SDRR—standard deviation of RR intervals; RRdif—mean of absolute differences between successive RR; CV—coefficient of variance; relRRdif = RRdif/(mean RR); mean RR—mean of RR intervals; RRrange = max(RR) − min(RR); relRRrange = RRrange/(mean RR) (relative RRrange).

Feature selection was conducted solely on the training set. One approach is selecting the features with the highest area under the receiver operating characteristic (ROC) curve (AUC). The AUCs of specific features are presented in Figure 1. pRR50 has the highest AUC, followed by relRRdif, RRdif, CV and relRRrange. However, selecting feature sets based only on AUC is not well suited when the features are correlated and carry redundant information.

**Figure 2:**
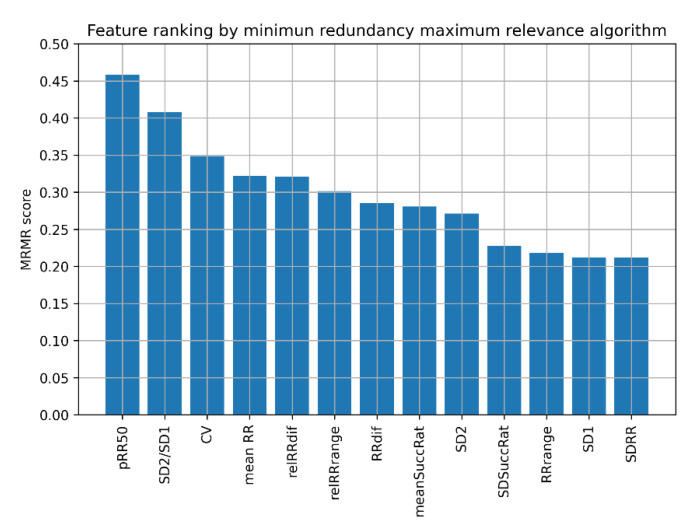
Ranking of heart rate variability features by minimum redundancy maximum relevance (MRMR) algorithm. pRR50—percentage of successive differences between RR intervals greater or equal to 50 ms; SD1 and SD2—standard deviation of points in the Poincare plot across and along the identity line, respectively; mean RR—mean of RR intervals; SDRR—standard deviation of RR intervals; RRdif—mean of absolute differences between successive RR; CV—coefficient of variance; relRRdif = RRdif/(mean RR); meanSuccRat—mean ratio of successive RR; SDSuccRat—standard deviation of ratios of successive RR; RRrange = max(RR) − min(RR); relRRrange = RRrange/(mean RR) (relative RRrange).

For this reason, we decided to use the MRMR algorithm to select the best feature sets for AF detection for ML algorithms. It is a filter-based feature selection algorithm [60], which orders the most relevant features providing minimal redundancy between subsequent features simultaneously. MRMR scores obtained for 60 s ECG recordings are presented in Figure 2.

The results show that the highest MRMR scores are obtained subsequently for pRR50, SD2/SD1, and CV, followed by mean RR, relRRdif, and relRRrange. Using these results, we examined the performance of different ML classifiers for different numbers of the best features determined by the MRMR algorithm.

**Figure 3:**
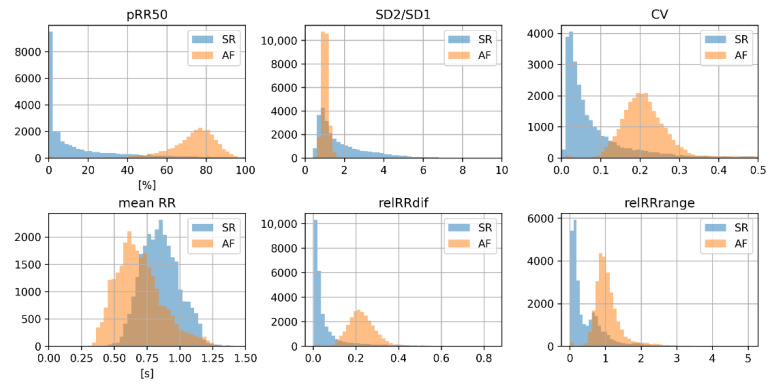
Histograms of six heart rate variability parameters with the highest score from minimum redundancy maximum relevance algorithm (blue—sinus rhythm, SR; orange—atrial fibrillation, AF). pRR50—percentage of successive differences between RR intervals greater or equal to 50 ms; SD1 and SD2—standard deviation of points in the Poincare plot across and along the identity line, respectively; CV—coefficient of variance; mean RR—mean of RR intervals; relRRdif—mean of absolute differences between successive RR divided by mean RR; relRRrange = (max(RR) − min(RR))/(mean RR) (relative RRrange).

To conclude, the relevance of particular HRV parameters in distinguishing between AF and SR (based on the results from MRMR) is not strictly related to their AUC. The distributions of features in the training set in AF and SR are presented as histograms in Figure 3. In general, histograms of the features for AF and SR have a smaller overlap in features with higher AUC (see Figure 1).

### 2.7. Classification Algorithms

The following standard classification algorithms were employed and compared in this study for AF detection:

- Decision Tree (DT),
- *K* Nearest Neighbors (KNN),
- Support Vector Machine with the linear kernel (SVM linear),
- Support Vector Machine with radial basis function kernel (SVM RBF),
- Ada Boost (ADA),
- Random Forest (RF),
- Artificial Neural Network (ANN).

In DT, a set of conditional statements (nodes) forming a tree are used for classification. Values of particular features are compared with threshold values in the nodes. During DT’s training (building), new nodes in the tree are added by choosing the feature that splits the tree best according to some metric. We used Gini impurity as a metric of split quality [61].

In KNN, classification is made by measuring the distances between a new sample (whose class is unknown) and all the training samples (with known classes). *K* samples with the smallest distances (nearest neighbors) are selected, and the most common class among them is chosen as the class of the new sample [62].

SVM is a classification algorithm in which a hyperplane is chosen as a decision boundary separating two classes. Ideally, entire classes should be on opposite sides of the hyperplane. Moreover, the minimal distance of the training examples from the hyperplane is maximized by SVM. If the classes are not linearly separable, the problem can be mapped to a higher dimension using a transform (kernel), such as Radial Basis Function (RBF) [63].

ADA is an ensemble learning method where classification is based on decisions from multiple simple classifiers. Training of the classifiers is sequential. For each classifier, the training set is modified by adjusting the weights of particular examples. The weight is increased if the example was incorrectly classified by the previous classifier and decreased otherwise. The final classification decision is a weighted majority vote of all classifiers [64].

RF is another ensemble learning method. Classifications from multiple decision trees are used as votes, and the most commonly voted class is used as the final classification decision. Each of the trees in the forest is built using a different subset of the training dataset [65].

ANN is a vast class of algorithms based on applying an artificial neural network concept that is also used in classification problems. We used the simplest feedforward ANN with one hidden layer and ReLU (rectified linear unit) activation function.

## 3. Results

**Figure 4:**
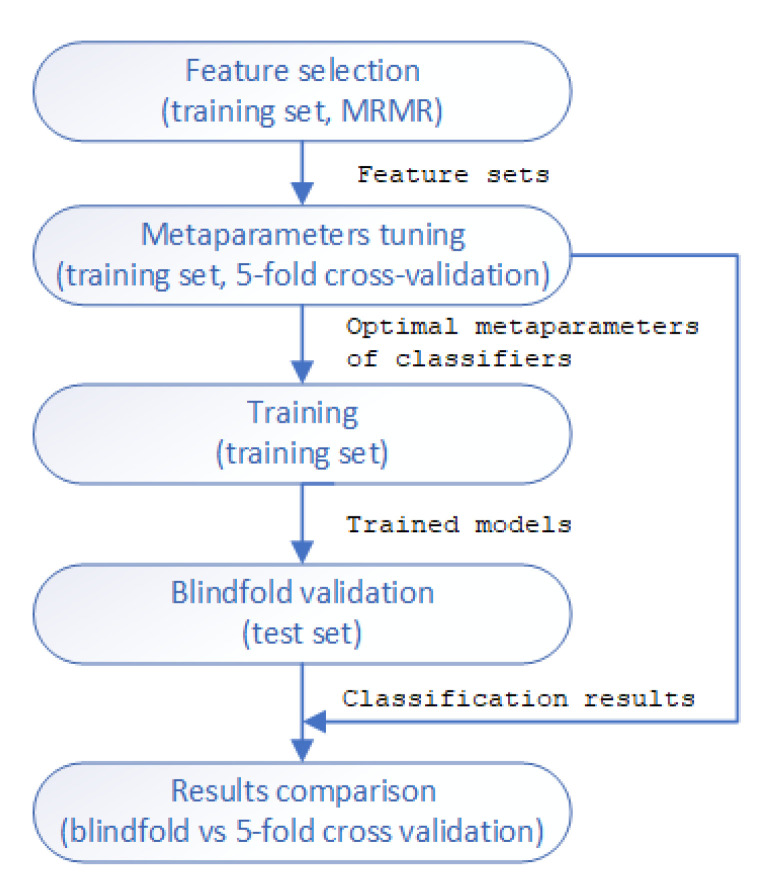
Diagram of training and validation methodology. MRMR—minimum redundancy maximum relevance.

The methodology of classifier training in the study is presented in Figure 4. First, MRMR was used for feature selection (on the training set).

Then, sets of one to six features with the highest MRMR scores were used for the metaparameter tuning of the classifiers (in case of DT—the maximum depth of the tree, in KNN—the number of neighbors K, in SVM with linear kernel—the soft margin C, in SVM with RBF kernel—both the soft margin C and inverse of kernel’s width gamma, in RF—the maximum depth and number of classifiers, in ADA—the number of classifiers, in ANN—the number of hidden neurons). The 5-fold cross-validation on the training set was used to find the best metaparameters of the classifiers. The metaparameters for which the highest average accuracy in the 5-fold cross-validation was achieved were chosen as the best for each feature set. Then, the classifiers with optimal metaparameters were trained on the training set, and their performance was validated on the test set. The results were then compared with the results obtained for the 5-fold cross-validation. We calculated the accuracy, sensitivity, specificity, positive predictive value (PPV), and diagnostic odds ratio (DOR) [66] of the classification. We decided to include DOR as a useful single metric in diagnostic testing. DOR is rarely reported in the literature on AF detection, with exceptions like [40].

### 3.1. Feature Sets with pRR50

**Figure 5:**
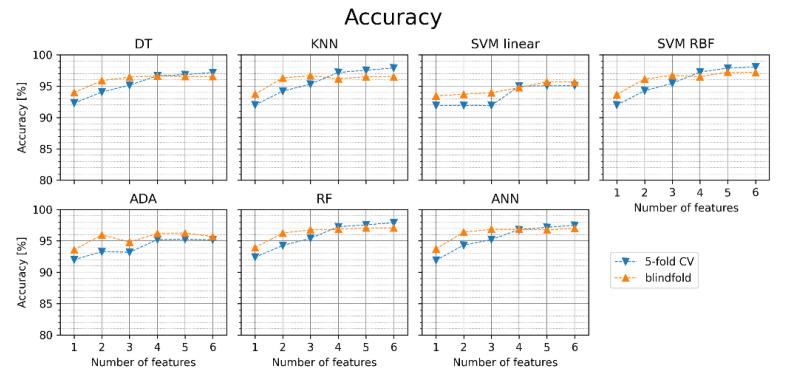
Accuracy of AF detection for different classifiers with sets of one to six features (orange points—blindfold validation, blue points—5-fold cross-validation). DT—decision tree, KNN—K nearest neighbors, SVM linear—support vector machine with linear kernel, SVM RBF—support vector machine with radial basis function kernel, ADA—Ada Boost, RF—random forest, ANN—artificial neural network.

**Figure 6:**
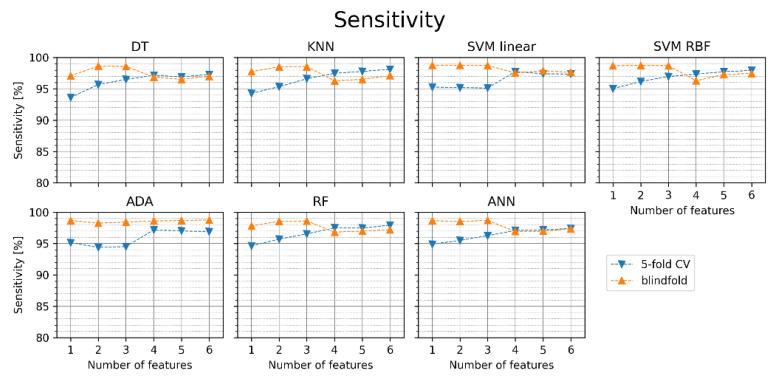
Sensitivity of atrial fibrillation detection for different classifiers with sets of one to six features (orange points—blindfold validation, blue points—5-fold cross-validation). DT—decision tree, KNN—K nearest neighbors, SVM linear—support vector machine with linear kernel, SVM RBF—support vector machine with radial basis function kernel, ADA—Ada Boost, RF—random forest, ANN—artificial neural network.

**Figure 7:**
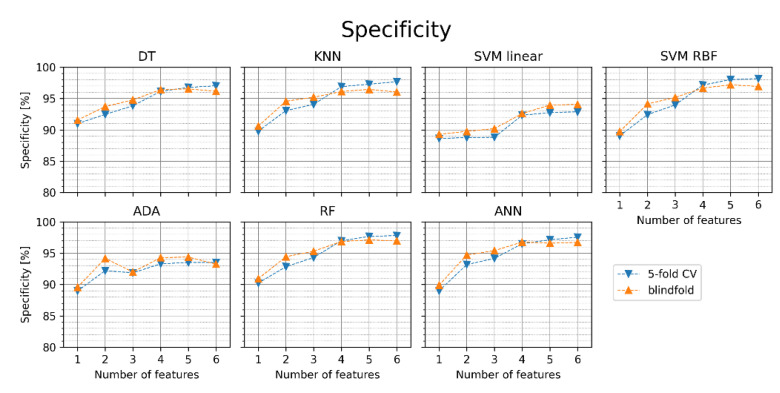
Specificity of AF detection for different classifiers with sets of one to six features (orange points—blindfold validation, blue points—5-fold cross-validation). DT—decision tree, KNN—K nearest neighbors, SVM linear—support vector machine with linear kernel, SVM RBF—support vector machine with radial basis function kernel, ADA—Ada Boost, RF—random forest, ANN—artificial neural network.

**Figure 8:**
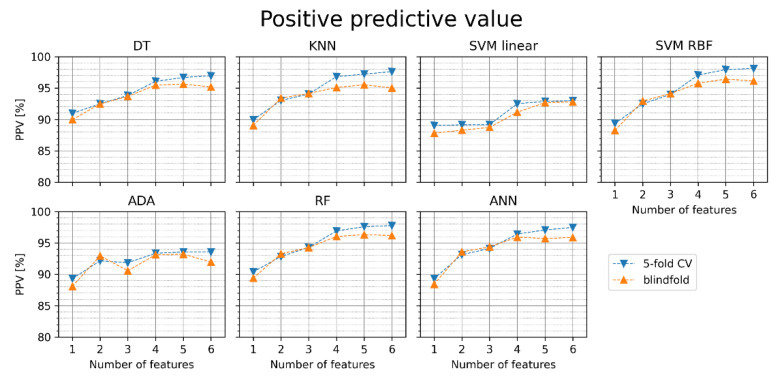
Positive predictive value (PPV) of AF detection for different classifiers with sets of one to six features (orange points—blindfold validation, blue points—5-fold cross-validation). DT—decision tree, KNN—K nearest neighbors, SVM linear—support vector machine with linear kernel, SVM RBF—support vector machine with radial basis function kernel, ADA—Ada Boost, RF—random forest, ANN—artificial neural network.

The classification metrics (accuracy, sensitivity, specificity, positive predictive value, and diagnostic odds ratio—DOR [66]) obtained by the ML classifiers in the 5-fold cross-validation and blindfold validation are presented in Figure 4, Figure 5, Figure 6, Figure 7 and Figure 8. For each classifier, one to six features with the highest MRMR scores were used for training, as summarized in Table 3.

**Figure 9:**
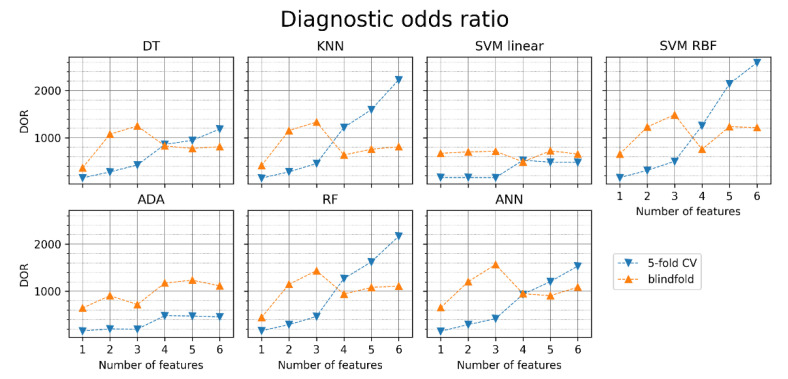
The diagnostic odds ratio (DOR) of AF detection for different classifiers with sets of one to six features (orange points—blindfold validation, blue points—5-fold cross-validation). DT—decision tree, KNN—K nearest neighbors, SVM linear—support vector machine with linear kernel, SVM RBF—support vector machine with radial basis function kernel, ADA—Ada Boost, RF—random forest, ANN—artificial neural network.

Figure 5 presents the accuracy obtained in our experiments. The orange points in the figure relate to the blindfold validation on the test set and the blue points to the 5-fold cross-validation. The same convention of presenting the results has been applied to other classification metrics in Figure 6, Figure 7, Figure 8 and Figure 9. Average values and standard deviations of accuracy are shown in Table 4. Standard deviations are small (below 0.5 pp), which is why similar tables are not included further in this paper for other classification metrics. Figure 4 indicates that for most ML classifiers, the increase in the number of features improves the accuracy of AF detection in both cases: for the cross-validation and the blindfold validation. In all classifiers, except for SVM linear and ADA, accuracy in blindfold validation drops below the 5-fold CV level for four-six features. The obtained results are better for DT, KNN, RF, SVM RBF, and ANN than for SVM linear and ADA. SVM RBF with five features achieved the highest accuracy in the blindfold validation (97.2%). The results suggest that using the simpler classification algorithms as DT and KNN can provide comparable or slightly worse accuracy and other performance metrics (see Figure 6, Figure 7, Figure 8 and Figure 9) than the relatively more complex algorithms such as SVM RBF, RF, or ANN.

Figure 6 shows the sensitivity for the particular classifiers obtained in the same experiments as the accuracy in Figure 5. The achieved sensitivity values are similar for all classifiers. In the 5-fold cross-validation, the increase in the number of features improves sensitivity, but most classifiers reach the maximum for three features in the blindfold validation. Similarly, as in the case of accuracy, the obtained sensitivities are often greater for the blindfold validation than for cross-validation. SVM linear with one feature achieved the highest sensitivity in the blindfold validation (98.8%).

In Figure 7, the specificity of the particular classifiers is presented. Interestingly, in many cases, the specificity is higher in the blindfold validation than in the 5-fold cross-validation. Increasing the number of features generally improves the results. SVM RBF achieved the highest specificity in the blindfold validation with five features (97.2%).

Figure 8 shows the positive predictive value (PPV) obtained for the particular classifiers. In the 5-fold cross-validation and blindfold validation, increasing the number of features increases PPV in most classifiers. SVM RBF achieved the highest PPV in the blindfold validation with five features (96.4%).

Figure 9 shows the diagnostic odds ratio obtained for the particular classifiers. We can observe how the odds of AF detection grow with the increased number of features for different classifiers. ANN with three features achieved the highest DOR in the blindfold validation (1566). Similarly to accuracy, DOR in blindfold validation is higher than in 5-fold CV for 1–3 features and lower for 4–6 features in all classifiers, except for SVM linear and ADA.

### 3.2. Feature Sets without pRR50

**Figure 10:**
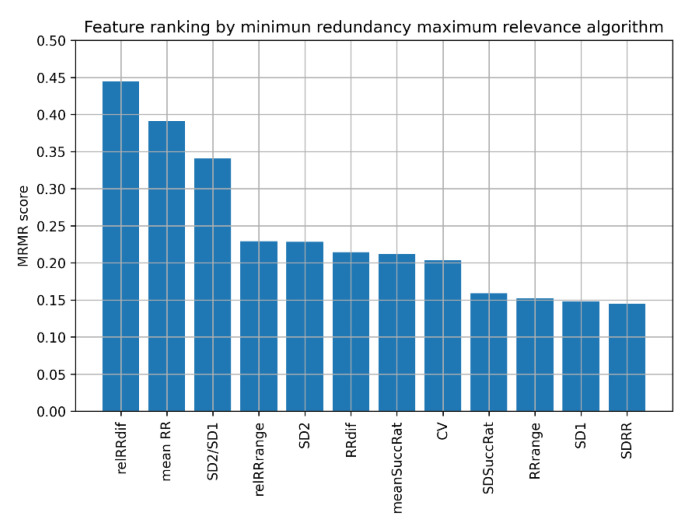
Ranking of heart rate variability features by minimum redundancy maximum relevance (MRMR) algorithm after excluding percentage of successive differences between RR intervals greater or equal to 50 ms (pRR50). SD1 and SD2—standard deviation of points in the Poincare plot across and along the identity line, respectively; mean RR—mean of RR intervals; SDRR—standard deviation of RR intervals; RRdif—mean of absolute differences between successive RR; CV—coefficient of variance; relRRdif = RRdif/(mean RR); meanSuccRat—mean ratio of successive RR; SDSuccRat—standard deviation of ratios of successive RR; RRrange = max(RR) − min(RR); relRRrange = RRrange/(mean RR) (relative RRrange).

In the next series of experiments, we repeated the feature selection process after excluding pRR50 from the analyzed features to verify how much diagnostic information was derived from pRR50, the highest scored parameter in MRMR. To provide the best comparable conditions, we repeated the MRMR analysis without pRR50, and the obtained MRMR scores are presented in Figure 10.

**Figure 11:**
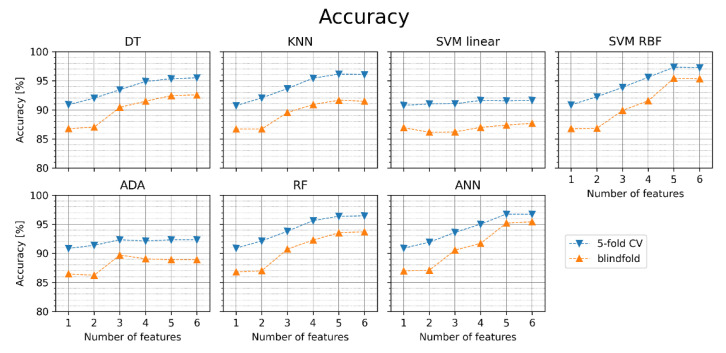
Accuracy of atrial fibrillation detection for different classifiers with sets of one to six features without pRR50 (orange points—blindfold validation, blue points—5-fold cross-validation). DT—decision tree, KNN—K nearest neighbors, SVM linear—support vector machine with linear kernel, SVM RBF—support vector machine with radial basis function kernel, ADA—Ada Boost, RF—random forest, ANN—artificial neural network.

The metaparameter tuning in the 5-fold cross-validation was repeated for new feature sets. The tuned classifiers were then trained and blindfold-validated. The accuracy obtained in this case is presented in Figure 11. Comparing Figure 11 with Figure 5, one can see the impact of the pRR50 parameter in AF detection procedures. SVM RBF achieved the highest accuracy in blindfold validation with five features (95.4%).

**Figure 12:**
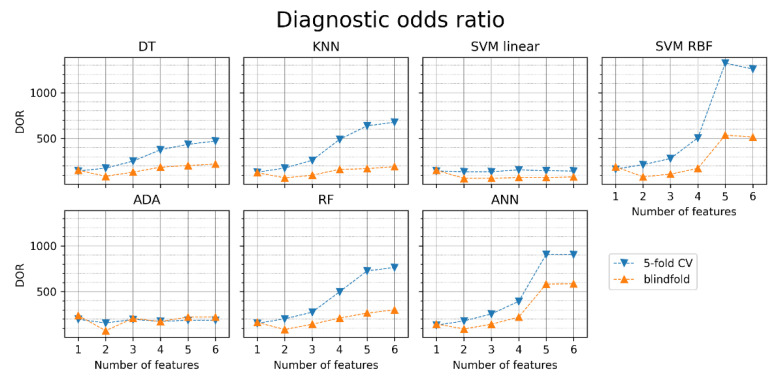
Diagnostic odds ratio (DOR) of atrial fibrillation detection for different classifiers with sets of one to six features (orange points—blindfold validation, blue points—5-fold cross-validation). DT—decision tree, KNN—K nearest neighbors, SVM linear—support vector machine with linear kernel, SVM RBF—support vector machine with radial basis function kernel, ADA—Ada Boost, RF—random forest, ANN—artificial neural network.

The diagnostic odds ratios obtained for different classifiers and different sets of features are presented in Figure 12. The obtained DORs are notably lower than when the pRR50 was included (see Figure 9). Without pRR50, the highest DOR in the blindfold validation was achieved by ANN with six features (585).

## 4. Discussion

Our findings demonstrate several things about discerning AF from SR using HRV parameters, selecting features for ML models, and different ML algorithms. The diagnostic properties of the applied ML algorithms are sensitive to the method used for choosing HRV parameters and the set of parameters entering the selection process. Feature selection based on AUC and MRMR gives different results. One might also see SR and AF histograms of various HRV parameters (Figure 3). Such a visual comparison shows which parameters may separate SR from AF. It is worth noting that for some features, such as mean RR and SD2/SD1, the distributions in AF and SR are not well separated, so these features are not good sole predictors of AF, but they bring additional information valuable in the presence of other features. Of the top six HRV features selected by MRMR, only four (66.7%) were in the top six with the highest AUC. In both methods, however, pRR50 was the number one HRV feature.

Furthermore, the analyzed HRV parameters describe distinguishing features of RR interval time series based on the absolute or relative differences between consecutive RR intervals, their ratios, and distributions. Interestingly, the most complex six-element MRMR-derived set mostly included the relative HRV parameters except for the mean RR.

In all six MRMR-selected feature sets, pRR50 was included. When pRR50 was excluded from the MRMR analysis, the order of selected parameters differed notably (see Figure 3 and Figure 10). ML algorithms always performed better with pRR50 than without pRR50, regardless of the number of features between 1 and 6 (as measured by blindfold validation DOR—see Figure 9 and Figure 12). For example, in SVM RBF with six features, the DOR was around 1200 when pRR50 was used and around 500 without pRR50.

We showed (Figure 2) which HRV parameters (features) contribute to the maximal diagnostic value, simultaneously providing minimal redundancy between subsequent features in AF detection. The 5-fold cross-validation (blue points in Figure 5, Figure 6, Figure 7, Figure 8, Figure 9, Figure 11 and Figure 12) confirmed the gradual growth of the statistical measures of AF detection with the increase in the number of the best HRV parameters chosen by the MRMR algorithm. Adding the subsequent sixth feature no longer causes a noticeable increase in the statistical measures, and in the case of some measures, even a very slight decrease. The blindfold validation results (orange points in Figure 5, Figure 6, Figure 7, Figure 8, Figure 9, Figure 11 and Figure 12) behave similarly for most algorithms. By increasing the number of features, especially from one to four, accuracy and sensitivity improved. Nevertheless, the blindfold validation results do not differ considerably from the results obtained through the 5-fold cross-validation, especially for the best algorithms.

Moreover, slightly worse results in accuracy for the blindfold validation are typical in many classification problems. The differences between the 5-fold cross-validation and blindfold validation are noticeably significant, but not in all cases; using a separate dataset for validation results in worse performance. SVM RBF achieved the highest accuracy in blindfold validation with five features (97.2%). On the other hand, ANN achieved the highest DOR in blindfold validation with three features (1566). However, it is worth noting that comparatively good results were also achieved with relatively computationally simple classifiers such as KNN or even DT, while the worst results were obtained for SVM linear and ADA.

Notably, the performance of the considered ML algorithms for AF detection is significantly higher when the feature set includes pRR50. Even if only pRR50 is used, very good diagnostic results are obtained (accuracy between 93.4 and 93.9%). In comparison, similar accuracy without pRR50 is achieved for at least four features (87.3–95.4%). Moreover, performance in blindfold validation is noticeably worse than in 5-fold cross-validation, which was not always the case when pRR50 was used.

The use of a specific length of the ECG, i.e., 60 s, limits to some extent our conclusions only to the ECG recordings or AF episodes of such a length. Our preliminary results (data not shown) with other lengths do not change the overall conclusions. Nevertheless, the impact of ECG length on the statistical measures of AF detection performance using HRV parameters selected by means of the MRMR algorithm requires further detailed investigations. It should also be noted that the goal of our research was not to study various feature selection algorithms and determine the best one which is a very general and complex task.

The MRMR algorithm proved useful and valuable in selecting HRV parameters with the potential to distinguish AF from SR in the 60 s ECGs. Three HRV parameters, i.e., pRR50, SD2/SD1, and CV, were ranked highest by MRMR for ML-based AF detection and pRR50 appears to outperform other HRV parameters for this task. It has the highest AUC, and the feature sets containing it achieve higher accuracies than those without it (see Figure 5 and Figure 11).

The proposed methods and results presented in the paper might contribute to developing practical AF detection solutions in miniature wearable, bio-patches, implantable devices, and hand-held single- or multi-lead ECG devices [3,67,68,69,70].

It should be, however, kept in mind that the newer modes of ECG acquisition have their technical limitations, which may impact the quality of the recorded ECG signal and its noise level. If ECG quality declines, then the noise level increases, and there are a couple of reasons for it. Even if silver/silver chloride (Ag/AgCl) hydrogel electrodes are attached to a patient, there is always sweating and skin cell necrosis—this problem is particularly important in very long ECGs lasting for several consecutive days. Different materials are used to produce ECG electrodes. Metal ECG electrodes are a part of hand-held devices or smart-watches. Textile, polymer graphene, or rubber electrodes are used in the bio-patches, chest straps, or ECG vests. However, the ECG signal is usually not as good as the specialized Ag/AgCl electrodes [68,71,72,73,74,75,76]. Finally, electrical skin properties, including skin-electrode impedance, differ between patients; it changes with age, amount of subcutaneous adipose tissue or fluid accumulation, or the presence of some diseases [68,72].

For an ECG recorded with good quality, HRV parameters combined with ML methods are valuable for their potential clinical use. The number of devices dedicated to long-term ECG monitoring increases as they are vital in transient AF detection [3,68,69,70]. In these solutions, computational and energy efficiency are of crucial importance. Therefore, using straightforward ML classifiers and a small set of adequately selected simple HRV parameters is advisable. Having the results presented in the paper, we can consciously, i.e., based on the quantitative numerical results, choose both the ML algorithm and the extremely small sets of simple HRV features needed to achieve the assumed AF detection performance.

Recent studies have shown that advanced computational methods such as artificial intelligence may predict AF using the 12-lead and even a single-lead ECG acquired in patients with sinus rhythm [77,78,79]. The possibility of foreseeing the disease dramatically changes our perspective and clinical potential. Zachi et al. have proposed that with the artificial intelligence tools and modeling applied to proper data, it is possible to select previvors, i.e., individuals who are still healthy but have a substantial risk of developing a disease in the future [77]. Using artificial intelligence and structural analysis of resting ECG, it is possible to identify previvors of AF and start preventive actions before this arrhythmia and its complications occur. It might save lives, reduces morbidity, and probably the cost of AF management.

Very recently, Sagnard A. et al. [5] have shown that HRV analysis (mainly reduced LF/HF and increased pNN50 and RMSSD) predicted the new-onset in-hospital AF in over 2000 survivors of acute myocardial infarction. Their study suggests that HRV features also identify previvors of AF. However, it is unknown whether the employment of ML or artificial intelligence algorithms to an ECG in patients without AF would translate into the prediction of AF and identification of its previvors.

If HRV parameters and ML techniques can be implemented for diagnostic purposes in mobile e-health technologies, then why not use them to predict AF before it even happens? We are convinced that it is possible and that studying such a concept deserves future investigations.

The current clinical use of HRV deserves a short comment. For many years, HRV has been demonstrated to predict total or various forms of mortality, mainly in survivors of myocardial infarction and heart failure patients [12,14,80,81,82]. However, the constant progress in the healthcare and management of patients after myocardial infarction and heart failure has substantially reduced mortality and improved the long-term prognosis. Nowadays, more patients receive quick myocardial reperfusion and modern pharmacological treatment. These are just some of the many reasons why HRV is no longer recommended for the mortality risk stratification in cardiac patients. Nevertheless, both patients who suffer from heart attacks and those with heart failure are at risk of future developing AF. If HRV helped identify AF previvors, i.e., people at risk of the new onset of this arrhythmia, it would translate into a great return of this method to clinical practice.

## 5. Conclusions

HRV parameters combined with ML techniques differentiate ECGs with AF from those in SR. However, methods used for choosing HRV features may impact the outcome of the ML algorithm. Using straightforward ML classifiers and the extremely small sets of simple HRV features, regardless of the features selection methods used (AUC or MRMR), pRR50 has consistently been selected at the top HRV parameter differentiating AF from SR in ECGs of 60 s duration. The proposed methodology and the presented results of the selection of HRV parameters have the potential to develop practical solutions and devices for automatic AF detection with minimal sets of simple HRV parameters.

## Figures and Tables

**Table 1:** Atrial fibrillation detection results from the literature.

Reference	Dataset	Accuracy [%]	Sensitivity [%]	Specificity [%]	Classifier	Notes
Parsi et al., 2021 [28]	Physionet Atrial Fibrillation Prediction Database	97.7	98.8	96.7	SVM (also MLP, RF, KNN)	5 min ECG segments, established and new HRV parameters, the distinction between SR and rhythm just before the onset of AF. 10-fold cross-validation (by the patient).
Biton et al., 2021 [29]	Telehealth Network of Minas Gerais (TNMG) database	-	59.6	95.3	RF	DNN features from a raw 12-lead ECG (7–10 s), morphology, HRV, EMR metadata. Prediction of developing AF within 5 years.
Zhu et al., 2021 [30]	13,354 short-term ECG segments	90.46	94.04	86.74	ANN	10 s ECG, seven classes (rhythms). Feature selection: correlation with label, MRMR, Fisher criterion.
Jiang et al., 2022 [32]	Own BCG database	94.50	96.70	92.62	SVM (also KNB, LR, RF, BT)	24 s BCG signal, MRMR feature selection, 10-fold CV
Oster et al., 2013 [34]	AFDB	-	92.7	94.2	RR entropy threshold	Length: 12 RR intervals, features: coefficient of sample entropy.
Mohebbi et al., 2008 [35]	MIT-BIH Arrhythmia Database	-	99.07	100	SVM	HRV from 32 RR, feature selection with LDA
Sepulveda-Suescun et al., 2017 [36]	AFDB	97.8	97.9	97.8	SVM	Poincare plot-based HRV. Only 226 AF and 264 SR segments.
Nguyen et al., 2018 [37]	2017 PhysioNet/Computers in Cardiology Challenge Database	95.15	-	-	SVM	30–60 s recordings, HRV
Mei et al., 2018 [38]	2017 PhysioNet/Computers in Cardiology Challenge Database	96.6	83.2	98.6	BT	30–60 s recordings, HRV, and frequency analysis
Pourbabaee et al., 2018 [40]	Physionet PAF prediction challenge database	91	-	-	KNN (and other classifiers)	Neural network-extracted features.
Faust et al., 2018 [41]	AFDB	99.77	99.87	99.61	RNN	Sequence of 100 RR intervals
Ma et al., 2020 [43]	MIT-BIH Arrhythmia Database	98.3	97.4	99.3	Shallow ANN	RR interval series as features, not specified how long.
Marsili et al., 2016 [44]	AFDB	98.44	97.33	98.67	Symbolic dynamics, threshold	Beat-by-beat classification based on RR intervals (symbolic dynamics).
Erdenebayar et al., 2019 [45]	AFDB, MIT-BIH Normal Sinus Rhythm Database	98.7	98.7	98.6	CNN	30 s segments, CNN features from raw ECG, training/test division not specified.
Mousavi et al., 2020 [46]	AFDB	82.41	90.53	79.54	Bidirectional RNN	5 s RR interval sequence
Faust el al., 2020 [47]	AFDB, LTAFDB	94			RNN	100 RR sequence, blindfold validation on LTAFDB

AF—atrial fibrillation, AFDB—MIT-BIH Atrial Fibrillation Database, ANN—artificial neural network, BCG—ballistocardiogram, BT—bagging tree, CNN—convolutional neural network, DNN—deep neural network, ECG—electrocardiogram, EMR—electronic medical record, HRV—heart rate variability, KNB—kernel naive Bayes, KNN—K nearest neighbors, LDA—linear discrimination analysis, LR—linear regression, LTAFDB—Long-Term Atrial Fibrillation Database, MLP—multilayer perceptron, MRMR—minimum redundancy maximum relevance, PAF—paroxysmal atrial fibrillation, RF—random forest, RNN—recurrent neural network, RR—distance between peaks of consecutive R-waves, SR—sinus rhythm, SVM—support vector machine.

**Table 2:** The number of electrocardiogram segments with atrial fibrillation (AF) and sinus rhythm (SR) in training and test sets before and after discarding the segments with artifacts.

Dataset	AF Total	AF Filtered	SR Total	SR Filtered
Training	27,630	26,464	27,823	27,311
Test	11,191	11,167	25,461	14,294

**Table 3:** Feature sets used for the classification.

No. of Features	Feature Set
1	pRR50
2	pRR50, SD2/SD1,
3	pRR50, SD2/SD1, CV
4	pRR50, SD2/SD1, CV, mean RR
5	pRR50, SD2/SD1, CV, mean RR, relRRdif
6	pRR50, SD2/SD1, CV, mean RR, relRRdif, relRRrange

pRR50—percentage of successive differences between RR intervals greater or equal to 50 ms; SD1 and SD2—standard deviation of points in the Poincare plot across and along the identity line, respectively; CV—coefficient of variance; mean RR—mean of RR intervals; relRRdif—mean of absolute differences between successive RR divided by mean RR; relRRrange = max(RR) − min(RR)/(mean RR) (relative RRrange).

**Table 4:** Average values and standard deviations of accuracy in 5-fold cross-validation in 60 s recordings (in percentages).

	Features	1	2	3	4	5	6
Classifier							
**DT**		92.29 (0.27)	94.06 (0.27)	95.15 (0.15)	96.65 (0.13)	96.84 (0.10)	97.16 (0.09)
**KNN**		92.00 (0.38)	94.20 (0.32)	95.32 (0.19)	97.18 (0.13)	97.53 (0.14)	97.90 (0.12)
**SVM linear**		91.91 (0.35)	91.93 (0.34)	91.92 (0.31)	95.00 (0.16)	95.04 (0.16)	95.08 (0.16)
**SVM RBF**		91.99 (0.34)	94.26 (0.31)	95.45 (0.11)	97.25 (0.07)	97.87 (0.13)	98.06 (0.10)
**ADA**		92.01 (0.35)	93.28 (0.40)	93.18 (0.37)	95.21 (0.23)	95.24 (0.26)	95.19 (0.23)
**RF**		92.40 (0.32)	94.26 (0.26)	95.42 (0.13)	97.24 (0.15)	97.57 (0.09)	97.88 (0.13)
**ANN**		91.89 (0.30)	94.32 (0.36)	95.20 (0.11)	96.79 (0.21)	97.16 (0.29)	97.48 (0.23)

DT—decision tree, KNN—K nearest neighbors, SVM linear—support vector machine with linear kernel, SVM RBF—support vector machine with radial basis function kernel, ADA—Ada Boost, RF—random forest, ANN—artificial neural network.

## Data Availability

MIT-BIH Atrial Fibrillation Database (AFDB), [50,51] and Long Term AF Database (LTAFDB) [51,52] were used in the study. They are available at https://physionet.org/content/afdb/1.0.0/ (accessed on 1 June 2022) and https://physionet.org/content/ltafdb/1.0.0/ (accessed on 1 June 2022), respectively.
